# Post-Traumatic Stress, Compassion Fatigue, and Psychological Well-Being Among Critical Care Nurses in Saudi Arabia: A Cross-Sectional Study

**DOI:** 10.3390/healthcare14091188

**Published:** 2026-04-28

**Authors:** Sarah A. AlAbdalhai, Ali Kerari, Sanaa Ghulman

**Affiliations:** 1 College of Nursing, King Saud University, Riyadh 11421, Saudi Arabia; 444203675@student.ksu.edu.sa; 2 Nursing Administration and Education Department, College of Nursing, King Saud University, Riyadh 11421, Saudi Arabia; 3 Nursing Research Unit, King Saud University Medical City, Riyadh 11421, Saudi Arabia; sghulman@ksu.edu.sa

**Keywords:** PCL-5, compassion fatigue, psychological well-being, critical care nurses, Saudi Arabia

## Abstract

**Highlights:**

**What are the main findings?**

- Critical care nurses experience high levels of post-traumatic stress disorder (PTSD) symptoms and moderate compassion fatigue, with psychological well-being compromised in a substantial proportion of participants.
- PTSD symptoms and compassion fatigue are strongly associated with poorer well-being, whereas demographic factors have limited influence compared with work-related stressors.

**What are the implications of the main findings?**

- The findings highlight the need for system-level rather than demographic-based interventions, as mental health risks are primarily driven by workplace exposure and organizational pressures.
- Integrating routine psychological screening, trauma-informed support, and improved staffing and workload management may help protect nurses’ well-being.

**Abstract:**

**Background:** Critical care nurses are frequently exposed to traumatic clinical events and occupational stress, increasing the risk of post-traumatic stress disorder (PTSD), compassion fatigue, and compromised psychological well-being. However, the interrelationships among these variables in Saudi Arabia remain unclear. This study investigated the associations between PTSD symptoms, compassion fatigue, and psychological well-being among critical care nurses. **Methods:** A descriptive cross-sectional study was conducted between October and December 2025 with 210 critical care nurses from the Eastern and Riyadh regions of Saudi Arabia. Data were collected using the PTSD Checklist for DSM-5 (PCL-5), the Professional Quality of Life Scale, and the WHO-5 Well-Being Index. Data analysis included descriptive statistics, *t*-tests, one-way analysis of variance, Pearson’s correlation coefficients, and multiple linear regression. **Results:** The mean PCL-5 score was 27.44, with 38.1% of participants meeting the cutoff for probable PTSD. Compassion fatigue was moderate. The mean WHO-5 score was 54.60, indicating moderate well-being, though a substantial proportion reported poor well-being. Psychological well-being was negatively correlated with both PTSD symptoms and compassion fatigue, while PTSD symptoms were strongly positively correlated with compassion fatigue. Both PTSD and compassion fatigue independently predicted lower well-being, explaining 21% of the variance. Sociodemographic variables were not significant predictors after adjustment. **Conclusions:** Critical care nurses experience moderate PTSD symptoms and compassion fatigue, adversely affecting psychological well-being. These findings underscore the interconnected nature of trauma-related distress and professional quality of life, highlighting the need for routine psychological screening, trauma-informed support, and resilience-focused interventions.

## 1. Introduction

The nursing profession plays a crucial role in healthcare systems worldwide, and its importance has become increasingly evident in Saudi Arabia owing to rapid population growth and ongoing healthcare transformation. Saudi Arabia’s Vision 2030 emphasizes strengthening healthcare services, particularly focusing on enhancing the attractiveness and sustainability of the nursing profession as a cornerstone of the healthcare workforce [1]. However, nurses in Saudi Arabia continue to face substantial work-related stressors that negatively affect their quality of life, quality of care, and professional retention.

Post-traumatic stress disorder (PTSD) is a psychiatric disorder that may occur following exposure to or witnessing traumatic events involving actual or threatened death, serious injury, or sexual violence [2]. Nursing professionals are particularly vulnerable to traumatic and high-stress work environments, which can adversely affect their psychological well-being and result in stress-related disorders, including PTSD [3]. Nurses working in critical care units, emergency departments, oncology settings, and trauma services are particularly vulnerable to psychological distress owing to sustained exposure to severe illness, traumatic events, and patient mortality, which have been consistently associated with higher risks of PTSD, compassion fatigue, and reduced well-being [4,5]. PTSD symptoms among nurses may compromise patient safety and the quality of care through impaired concentration, increased medication errors, emotional detachment, and burnout [3]. PTSD is frequently accompanied by anxiety and depression, which further exacerbate occupational stress.

Globally, the prevalence of PTSD in the general population is approximately 3.9%, with trauma exposure affecting approximately 5.6% of individuals [6]. Healthcare workers have substantially higher rates of PTSD. In Saudi Arabia, a national cross-sectional study conducted during the COVID-19 pandemic reported PTSD prevalence rates ranging from 19.6% to 24.8%, with higher rates among female participants [7]. Further research conducted in the Riyadh region revealed that 33.4% of emergency department staff met the criteria for PTSD, with nurses and paramedics scoring higher than physicians on PTSD symptom scales [8]. Similarly, a 2022 study conducted in King Abdulaziz Medical City found that 26.7% of emergency medical personnel met the diagnostic criteria for PTSD [7].

In addition to trauma exposure, nurses frequently experience disrupted sleep patterns related to shift work, on-call duties, irregular nutrition, limited physical activity, and chronic fatigue. These occupational factors have been consistently associated with adverse physical health outcomes, including weight gain, gastrointestinal disturbances, metabolic dysregulation, and reduced physical functioning, which may compromise professional performance and the quality of care delivery [9].

Compassion is a fundamental professional value in nursing and is widely regarded as a core attribute of high-quality nursing practice. It encompasses empathy, kindness, patience, and a genuine desire to alleviate human suffering [10]. However, sustained emotional exposure to patient suffering may diminish nurses’ capacity for compassionate presence, resulting in mechanical task performance without emotional engagement. This process often leads to compassion fatigue, which is characterized by emotional, physical, and spiritual exhaustion [10].

Compassion fatigue is particularly prevalent among nurses working in psychiatric, oncological, and critical care settings, where patients often require intensive physical, psychological, and emotional support. Compassion fatigue manifests through two primary components: burnout, which is marked by emotional exhaustion, frustration, and disengagement, and secondary traumatic stress, which reflects trauma-related symptoms arising from indirect exposure to patient suffering. A meta-analysis of 21 international studies reported that up to 52.55% of nurses experience compassion fatigue, with prevalence varying widely across clinical settings. High levels of compassion fatigue are associated with reduced job satisfaction, poor mental and physical health, increased medication errors, suboptimal patient care, and elevated staff turnover [11].

Both PTSD and compassion fatigue highlight the importance of nurses’ psychological and physical well-being. Psychological well-being includes life satisfaction, emotional balance, and the ability to adapt to adverse life experiences, all of which contribute to resilience and the protection of mental health [12]. Physical well-being encompasses fatigue levels, sleep quality, physical functioning, and health-promoting behaviors such as exercise, nutrition, and adequate rest. Nurses’ well-being is influenced by both their personal health status and the demands of caregiving roles [9,13]. Patient-related factors, including disease severity, functional impairment, and dependency, are also closely associated with nurses’ well-being [14]. Although well-being is a multidimensional construct encompassing both physical and psychological domains, the present study specifically focuses on psychological well-being, as measured by the WHO-5 Well-Being Index.

Despite growing international evidence, research in Saudi Arabia has largely focused on the prevalence of PTSD, and its relationship with compassion fatigue and overall well-being among critical care nurses remains unclear. A significant gap persists in understanding how PTSD and compassion fatigue jointly influence nurses’ psychological well-being in the Saudi healthcare context. Addressing this gap is essential for developing targeted interventions that support nurses’ resilience, improve the quality of patient care, and enhance workforce sustainability. Therefore, this study aimed to examine the relationships between PTSD, compassion fatigue, and well-being among critical care nurses in Saudi Arabia. Additionally, it sought to explore differences in these variables based on sociodemographic characteristics and to assess the effect of PTSD and compassion fatigue on nurses’ well-being after controlling for relevant demographic factors. The following research questions guided this study:

1. What are the relationships between PTSD, compassion fatigue, and psychological well-being among critical care nurses in Saudi Arabia?
2. Are there differences in post-traumatic stress, compassion fatigue, and psychological well-being based on the sociodemographic characteristics of critical care nurses?
3. Do PTSD and compassion fatigue affect the psychological well-being of critical care nurses after controlling for sociodemographic factors?

To address these research questions, the study objectives were formulated to reflect the key analytical components of the investigation. The objectives of this study were to:

1. Examine the relationships between post-traumatic stress, compassion fatigue, and psychological well-being among critical care nurses in Saudi Arabia.
2. Assess differences in post-traumatic stress, compassion fatigue, and psychological well-being across the sociodemographic characteristics of critical care nurses.
3. Determine the extent to which post-traumatic stress and compassion fatigue predict psychological well-being after controlling for sociodemographic factors.

In addition, the current study formulated the following hypotheses:

**H1.**  *Post-traumatic stress and compassion fatigue will be positively associated with each other, and both will be negatively associated with psychological well-being among critical care nurses in Saudi Arabia.*

**H2.**  *Levels of post-traumatic stress, compassion fatigue, and psychological well-being will differ significantly according to selected sociodemographic characteristics of critical care nurses.*

**H3.**  *Post-traumatic stress and compassion fatigue will significantly predict psychological well-being after controlling for sociodemographic factors.*

## 2. Materials and Methods

### 2.1. Study Design

This descriptive, cross-sectional study was conducted between October and December 2025. This design was selected to examine the relationships between PTSD, compassion fatigue, and well-being among critical care nurses, as well as their associations with selected demographic and work-related variables. This approach was considered appropriate owing to its feasibility and its ability to address the study objectives within a limited timeframe.

### 2.2. Setting and Sample

The study population comprised registered male and female nurses working in critical care units in public hospitals across the Eastern, Central (Riyadh), and Western regions of Saudi Arabia. Convenience sampling was used to recruit critical care nurses from intensive care units (ICUs), emergency departments, oncology wards, burn units, and hemodialysis units. The eligibility criteria were as follows: (1) being a registered nurse, (2) having at least one year of experience in a critical care setting, and (3) being able to read and understand English. The inclusion criteria were presented before access to the survey. Nurses with less than one year of critical care experience, those not currently working in critical care units, and those unable to comprehend English were excluded.

Sample size estimation was conducted using G*Power version 3.1.9.6 to ensure adequate statistical power. Assuming a significance level of α = 0.05, a power of 0.80, and a medium effect size (f^2^ = 0.15), the minimum required sample size for multiple linear regression analysis was calculated to be 143 participants. An additional 20–25% was added to account for potential non-response or incomplete surveys. Ultimately, 210 participants were recruited, exceeding the calculated sample size and thereby increasing the robustness and generalizability of the findings.

### 2.3. Ethical Considerations

Ethical approval was obtained from the Institutional Review Board of King Saud University (Ref No.: KSU-HE-25-963 approval dated 14 September 2025). Additional approval was obtained from the participating hospitals when required. Participation was voluntary, and eligible nurses were informed of their right to withdraw at any time without any consequences. Nurses provided online informed consent. Anonymity and confidentiality were strictly maintained, and all data were securely stored and accessible only to the research team.

### 2.4. Data Collection

Data were collected using an online questionnaire developed through Google Forms, which allowed participants to complete the survey via smartphones or tablets. Following ethical approval, the researchers coordinated with Health Clusters under the Ministry of Health in the Eastern, Central, and Western regions of Saudi Arabia to obtain permission for participant recruitment. Additional institutional approval was obtained from hospital Institutional Review Boards where required. The survey link was distributed through professional WhatsApp groups, and printed posters containing QR codes were displayed in the participating critical care units. The first page of the questionnaire included an electronic informed consent form, followed by demographic questions and standardized measurement instruments.

### 2.5. Measurements

The survey was administered in English, a professional language widely used by healthcare providers in Saudi Arabia. The questionnaire comprised demographic variables (age, sex, income, marital status, education level, years of experience, unit type, shift length, region, and patient-to-nurse ratio) and three validated instruments.

PTSD symptoms were assessed using the PTSD Checklist for DSM-5 (PCL-5), developed by the U.S. Department of Veterans Affairs [15]. The PCL-5 has demonstrated excellent internal consistency (α = 0.94) and good test–retest reliability (0.82) [3,15,16]. The instrument consists of 20 items representing four symptom clusters: intrusion (five items), avoidance (two items), negative alterations in cognition and mood (seven items), and alterations in arousal and reactivity (six items). Items were rated on a 5-point Likert scale ranging from 0 (“not at all”) to 4 (“extremely”). The total score was obtained by summing responses across all 20 items, yielding a possible range of 0 to 80. A score of 31 or higher was used as the threshold for identifying probable PTSD. Higher scores indicate greater severity of PTSD symptoms. In this study, the PCL-5 demonstrated excellent internal consistency (α = 0.95).

Professional quality of life was measured using the Professional Quality of Life Scale Version 5 (ProQOL V5) [17]. The ProQOL V5 comprises 30 items divided equally into three subscales: compassion satisfaction, burnout, and secondary traumatic stress. Participants rated the frequency of their work-related experiences over the previous 30 days using a 5-point Likert scale ranging from 1 (“never”) to 5 (“very often”). Each subscale yields a separate score ranging from 10 to 50, with higher scores indicating higher levels of the respective constructs. Previous studies conducted in Saudi Arabia have confirmed the scale’s reliability and cultural applicability, with Cronbach’s alpha values exceeding 0.70 across all subscales [10,18]. In the present study, internal consistency for the ProQOL was acceptable (α = 0.74).

Psychological well-being was assessed using the WHO-5 Well-Being Index, a widely validated instrument developed by Per Bech in the 1990s for measuring subjective mental well-being. This tool has been published as an open-access instrument by the WHO [19].

The WHO-5 consists of five positively worded items assessing well-being over the previous two weeks. Responses were rated on a 6-point Likert scale ranging from 0 (“at no time”) to 5 (“all of the time”), yielding a total score between 0 and 25. Raw scores were multiplied by four to obtain a final score ranging from 0 to 100, with scores ≤ 50 indicating poor well-being. The WHO-5 has demonstrated strong psychometric properties across diverse populations, with reported Cronbach’s alpha values ranging from 0.83 to 0.93 [19]. In this study, the instrument demonstrated excellent internal consistency (α = 0.90).

The selection of these instruments was guided by their strong psychometric properties, including established reliability and validity across diverse healthcare populations, as well as their widespread use in international research [3,15,16,17,19]. This enhances the comparability of the findings and strengthens the methodological rigor of the study.

### 2.6. Statistical Analysis

Data were exported from Google Forms into IBM SPSS Statistics version 29 for analysis. Prior to analysis, data cleaning was performed to ensure accuracy and completeness. This process included screening for missing data, duplicate entries, and ineligible responses based on predefined inclusion criteria. Cases with substantial missing data or failure to meet eligibility criteria were excluded from the final dataset. Descriptive statistics were computed to summarize participant characteristics and study variables, including frequencies and percentages for categorical variables, and means and standard deviations for continuous variables. The normality of continuous variables was assessed using skewness and kurtosis values.

Inferential analyses were conducted to examine group differences and relationships among variables. Independent-samples *t*-tests and one-way analysis of variance (ANOVA) were used to assess differences in PCL-5, compassion fatigue, and psychological well-being across demographic groups. When ANOVA results were statistically significant, post hoc comparisons were conducted to identify specific group differences. Pearson’s correlation coefficient was used to examine the strength and direction of associations between continuous variables. Multiple linear regression analysis was then performed to identify significant predictors of psychological well-being while controlling for relevant demographic variables. Regression coefficients (β), standard errors, and significance levels were reported. All assumptions for parametric tests were met. The internal consistency reliability of each instrument was evaluated using Cronbach’s alpha coefficients. Statistical significance was set at *p* < 0.05 for all analyses.

## 3. Results

### 3.1. Participant Demographics

Table 1 presents the sociodemographic and professional characteristics of the study participants (*n* = 210). Most participants were aged 25–34 years (*n* = 127, 60.5%), followed by those aged 35–44 years (*n* = 53, 25.2%); 30 participants (14.3%) were aged 45 years and above. Most respondents were female (*n* = 176, 83.8%), and over half were married (*n* = 124, 59.0%).

Most respondents held a bachelor’s degree (*n* = 158, 75.2%), followed by a master’s degree (*n* = 31, 14.8%). A small proportion held a diploma (*n* = 17, 8.1%) or a doctoral degree (n = 4, 1.9%). Clinical placement was predominantly in ICUs (*n* = 128, 61.0%), whereas others worked in coronary care, emergency, oncology, and burn units (*n* = 82, 39.0%). More than one-third of the participants had 10 or more years of professional experience (*n* = 78, 37.1%), followed by 3–5 years (*n* = 57, 27.1%) and 6–10 years (*n* = 39, 18.6%). A 12 h shift system accounted for the majority of work schedules (*n* = 141, 67.1%), whereas 49 participants (23.3%) worked 8 h shifts, and 20 (9.6%) reported other shift arrangements. Most nurses reported caring for one to two patients per shift (*n* = 103, 49.0%).

Geographically, more than half of the participants were employed in the Eastern region of Saudi Arabia (*n* = 121, 57.6%), whereas the remaining participants were employed in the Riyadh region (*n* = 89, 42.4%). Most respondents reported monthly incomes between 8000 and 12,000 Saudi Riyals (*n* = 95, 45.2%), followed by those earning more than 12,000 Saudi Riyals (*n* = 44, 20.9%).

### 3.2. PCL-5

The mean PTSD score based on the PCL-5 was 27 ± 17.5 (Table 2). Based on the PCL-5 cutoff score, 38.1% of critical care nurses were classified as having probable PTSD, whereas 61.9% were classified as not having probable PTSD. Independent-samples *t*-tests and one-way ANOVA indicated no significant differences between PCL-5 scores and sociodemographic variables.

### 3.3. Compassion Fatigue

Critical care nurses experienced moderate levels of both positive and negative dimensions of professional quality of life over the past 30 days. The mean burnout score (27 ± 5.3) indicates a moderate level of burnout, reflecting emotional exhaustion and work-related stress. The secondary traumatic stress score (26 ± 7.5) also falls within the moderate range, suggesting that nurses were experiencing noticeable, though not extreme, stress responses related to indirect exposure to patients’ traumatic experiences. Combined, the compassion fatigue score (53 ± 11.4) indicates a moderate overall burden of compassion fatigue, reflecting the cumulative impact of burnout and secondary traumatic stress (Table 2).

An independent-samples *t*-test examining differences by marital status revealed significant differences in compassion fatigue (Table 3). Married and unmarried participants differed significantly in terms of compassion fatigue (t [208] = 2.28, *p* = 0.024). However, no significant differences in compassion fatigue were observed across other sociodemographic variables (e.g., education, nursing shifts, work units, and work region).

Significant group differences in compassion fatigue were observed across income levels (F [3, 209] = 6.70, *p* < 0.01). Post hoc comparisons revealed significant differences between income groups, with participants reporting an income of more than 12,000 Saudi Riyals demonstrating higher compassion fatigue scores than other groups. Additionally, the nurse-to-patient ratio showed a significant difference in compassion fatigue scores (F [3, 209] = 3.19, *p* = 0.025). Post hoc testing confirmed significant mean differences between groups, with nurses caring for more than four patients reporting higher levels of compassion fatigue than those managing three or fewer patients.

### 3.4. Psychological Well-Being

The WHO-5 scores indicated that 58.1% of nurses reported moderate psychological well-being, with a mean score of 55.0 (standard deviation = 23.2). A significant difference was observed in psychological well-being according to marital status. Married participants reported higher scores on the Well-Being Index than unmarried participants (t [208] = 3.63, *p* < 0.001).

A one-way ANOVA revealed significant differences in psychological well-being across income levels (F [3, 209] = 3.76, *p* = 0.012). Post hoc analyses demonstrated significant mean differences between income groups, with nurses in higher-income categories reporting lower well-being scores. Professional experience was also significantly associated with psychological well-being (F [3, 209] = 9.83, *p* < 0.001). Post hoc comparisons indicated significant differences between professional experience groups, particularly among nurses with 1–2 years of experience. No significant associations were observed between psychological well-being and other sociodemographic variables.

### 3.5. Correlation Analyses of PTSD, Compassion Fatigue, and Psychological Well-Being

A significant negative correlation was observed between PTSD and psychological well-being (Table 4). Participants with higher PCL-5 scores reported lower levels of psychological well-being (r = −0.39, *p* < 0.001). Similarly, higher compassion fatigue scores were negatively correlated with psychological well-being (r = −0.40, *p* < 0.001). In contrast, a strong positive correlation was observed between PTSD and compassion fatigue (r = 0.71, *p* < 0.001), indicating that participants with higher levels of compassion fatigue also reported higher PCL-5 scores.

### 3.6. Effect of PCL-5 and Compassion Fatigue on Psychological Well-Being

Multiple regression analysis was conducted to assess the effect of compassion fatigue and PCL-5 on psychological well-being while accounting for other independent variables in the model (Table 5). The model demonstrated a good fit (F [8, 201] = 17.03, *p* < 0.01) and explained 21% of the variance in psychological well-being. Compassion fatigue and PCL-5 were significant predictors of psychological well-being (β = −0.20, 95% confidence interval [CI] = −0.191 to −0.010, *p* < 0.05; β = −0.22, 95% CI = −0.133 to −0.015, *p* < 0.05, respectively), after adjusting for demographic variables. These findings suggest that participants with higher levels of compassion fatigue or post-traumatic stress exhibited lower levels of psychological well-being. However, no significant effects of sociodemographic variables on psychological well-being were observed.

## 4. Discussion

This study showed that critical care nurses are at an increased risk of trauma-related psychological distress owing to repeated exposure to critically ill patients, frequent deaths, and sustained high-intensity workloads. Overall, the findings reveal a complex interplay between post-traumatic stress symptoms, compassion fatigue, and well-being, highlighting both resilience and vulnerability within this essential workforce. These findings may be valuable for improving the well-being of nurses who work with critically ill patients through regular quarterly assessments for manifestations of PTSD and compassion fatigue.

At the national level, AlAteeq et al. [20] and Alharbi et al. [21] reported elevated levels of psychological distress, including PTSD-related symptoms, anxiety, and stress, among nurses working in critical and emergency care settings, particularly under conditions of prolonged workload pressure and occupational stress. However, in this study, no significant differences in PTSD symptoms were observed across sociodemographic or professional variables, including education level, geographic region, marital status, shift length, age, income, years of experience, or nurse-to-patient ratio. These findings suggest that PTSD symptoms are widely distributed across demographic subgroups, indicating that exposure-related occupational stressors may outweigh individual demographic characteristics in shaping trauma-related outcomes among critical care nurses.

Instead, PTSD symptoms appear to be more strongly influenced by cumulative exposure to critical incidents and work-related trauma, regardless of age group [4,22]. Similar findings have been reported in systematic and scoping reviews, emphasizing that repeated exposure to high-acuity clinical situations may homogenize the risk of PTSD across age categories in intensive care settings [22].

Likewise, the lack of significant differences in PTSD symptoms by educational level, marital status, and geographic region is consistent with the contemporary literature, suggesting that formal educational attainment and personal demographic characteristics offer limited protection against trauma-related psychological distress in high-intensity clinical environments. Recent studies among frontline and ICU nurses have shown that PTSD symptoms are primarily driven by workplace trauma and organizational stressors rather than sociodemographic background [12,23]. Furthermore, the absence of significant associations between PTSD symptoms and professional factors such as years of experience, shift length, and nurse-to-patient ratio may reflect the pervasive nature of trauma exposure in critical care contexts.

In this study, critical care nurses experienced moderate levels of compassion fatigue. This finding is consistent with recent international studies reporting moderate levels of compassion fatigue among ICU nurses, even during non-crisis periods, highlighting compassion fatigue as a chronic occupational risk rather than a transient response [18,24]. The absence of significant associations between compassion fatigue and educational level, geographic region, and shift length suggests that compassion fatigue is not strongly influenced by demographic characteristics alone. Instead, these outcomes appear to be more closely associated with workload-related and psychosocial factors, as supported by recent evidence emphasizing organizational and environmental determinants of professional quality of life [5]. Marital status was a significant factor associated with compassion fatigue. This finding aligns with a previous study indicating that social and familial support can serve as a protective resource against compassion fatigue [25]. Conversely, limited social support or competing family demands may exacerbate emotional exhaustion, highlighting the importance of contextual and relational factors in shaping professional quality of life.

Additionally, significant differences in compassion fatigue were observed across income levels, years of professional experience, and nurse-to-patient ratios. Lower income, heavier patient loads, and imbalanced staffing ratios are associated with higher levels of compassion fatigue, driven by increased workload pressure and reduced perceived organizational support [13,18]. Similarly, although professional experience can enhance coping capacity, prolonged exposure to emotionally demanding environments may also increase fatigue, which may explain the differences observed across experience groups [24].

The WHO-5 findings of the present study indicate a heterogeneous profile of well-being among critical care nurses. Although more than half of the participants reported good well-being, a substantial proportion reported poor well-being, with a mean WHO-5 score of 55. The absence of significant differences in well-being according to educational level, geographic region, marital status, and shift length suggests that participants’ demographic characteristics do not strongly determine psychological well-being. Instead, well-being appears to be more closely associated with income, as evidenced by the significant differences across income groups. Similar results have been reported in recent international studies demonstrating that lower income and perceived financial strain are independently associated with poorer mental well-being among nurses, likely owing to increased stress, reduced job satisfaction, and limited supportive resources [26,27].

These findings highlight the interconnected nature of trauma-related distress, compassion fatigue, and psychological well-being in critical care nurses. Rather than operating as isolated phenomena, PTSD symptoms and compassion fatigue appear to form a mutually reinforcing cycle that places nurses at a heightened risk of compromised mental health. This pattern supports existing conceptualizations of professional quality of life, which view emotional exhaustion and secondary traumatic stress as closely intertwined with trauma exposure in high-intensity clinical environments [4,22].

Similarly, PTSD symptoms were strongly associated with poorer psychological well-being, reinforcing the notion that unresolved trauma-related distress plays a pivotal role in shaping nurses’ mental health trajectories. When combined with insufficient recovery opportunities, exposure to traumatic clinical events may contribute to depression and cognitive distress, all of which negatively influence overall well-being.

PTSD and compassion fatigue emerged as negative predictors of psychological well-being. Collectively, these findings support theoretical models of professional quality of life that conceptualize PTSD and compassion fatigue as interconnected risk pathways. The results highlight the importance of interventions that not only reduce exposure to traumatic stressors but also actively promote professional fulfillment, emotional support, and healthy work environments. Addressing compassion fatigue and PTSD concurrently, while strengthening protective factors, may be essential for sustaining psychological well-being among critical care nurses.

The findings of this study have important implications for clinical practice, organizational leadership, and health policy, particularly within the context of healthcare system transformation in Saudi Arabia. Given the high prevalence of PTSD symptoms, moderate but potentially fragile professional quality of life, and suboptimal well-being among a substantial proportion of critical care nurses, targeted and systemic interventions are warranted.

### 4.1. Practical Implication

At the clinical level, routine psychological screening should be integrated into occupational health and nursing support programs, especially in high-acuity environments such as ICUs. Brief and validated instruments, including the PCL-5, ProQOL, and WHO-5 Well-Being Index, have been widely recommended for the regular monitoring of healthcare workers’ mental health because of their feasibility and strong psychometric properties [3,20]. Early identification of PTSD symptoms, burnout, or reduced well-being enables timely referral to mental health services and preventive interventions, thereby reducing the risk of symptom escalation and long-term psychological impairment [22,28].

Additionally, early-career and newly hired nurses may require particular attention, as limited clinical experience is associated with reduced coping capacity and higher vulnerability to occupational stress in critical care settings [5,12]. Structured onboarding programs that incorporate resilience training, mentorship, and psychological support may be particularly beneficial during the early stages of professional practice.

Moreover, trauma-informed care principles should extend beyond patients to include healthcare providers. For instance, structured debriefings following traumatic clinical events, peer support initiatives, and access to confidential counseling services may help nurses process distressing experiences more effectively [22]. Such approaches acknowledge the cumulative nature of workplace trauma and foster a culture of psychological safety within healthcare organizations. Sleep disruption and chronic fatigue are strongly associated with poor psychological well-being, impaired functioning, and an increased risk of burnout and mental health problems among nurses [9]; therefore, promoting adequate sleep, rest breaks, and recovery time, particularly for shift workers, is essential.

### 4.2. Theoretical Implication

These findings have important theoretical implications within the context of Saudi Arabia’s Vision 2030, which emphasizes enhancing quality of life, strengthening healthcare system performance, and safeguarding workforce well-being as core national priorities. The results suggest that psychological well-being among critical care nurses should be conceptualized within integrated theoretical frameworks that link trauma exposure, compassion fatigue, and organizational stressors. Future research is needed to develop and test theory-driven models, such as self-efficacy and resilience-based frameworks, to better explain and enhance nurses’ well-being in high-acuity settings. In addition, applying and extending PTSD-related theoretical models within the nursing context may provide a deeper understanding of how trauma-related processes influence clinical performance, patient safety, and the overall healthcare environment in Saudi Arabia. A theoretically grounded approach is essential to guide evidence-based interventions that promote both individual- and system-level resilience in critical care settings.

### 4.3. Limitation

This study has several limitations. The cross-sectional design limits causal inference, and self-reported measures may be subject to reporting bias. The study utilized a convenience sampling approach, which may introduce self-selection and response biases. Nurses who chose to participate may differ systematically from those who did not, particularly in relation to their psychological well-being or interest in the study topic. As a result, the findings may be subject to potential overestimation or underestimation of the measured outcomes. Additionally, the use of non-probability sampling limits the generalizability of the results beyond similar clinical settings. Therefore, the findings should be interpreted with caution. Consequently, longitudinal and mixed-methods studies are recommended to explore changes in psychological outcomes over time and to evaluate the effectiveness of organizational interventions. Future research should focus on culturally tailored resilience and mental health programs that align with national priorities and nursing workforce needs.

However, this study also has several strengths that enhance the relevance of its findings. First, the study employed validated and widely used psychometric instruments, including the PTSD Checklist for DSM-5 (PCL-5), the Professional Quality of Life Scale (ProQOL), and the WHO-5 Well-Being Index, ensuring reliability, validity, and comparability with international research. Second, the inclusion of a relatively large and clinically relevant sample of critical care nurses strengthens the generalizability of the findings within similar healthcare settings. Third, this study addresses an important gap in the literature within the Saudi Arabian and Gulf context, where empirical data on post-traumatic stress, compassion fatigue, and well-being among critical care nurses remain limited. By focusing on this population, the study contributes context-specific evidence aligned with ongoing healthcare transformation efforts, including those related to workforce well-being.

## 5. Conclusions

The findings demonstrate a significant psychological burden, as 38.1% of nurses met the cutoff criteria for probable PTSD. Notably, professional experience emerged as a protective factor, suggesting that adaptation and coping capacities may improve over time. Conversely, higher PTSD symptom severity is strongly associated with poorer well-being, highlighting the urgent need for early detection and timely intervention. Healthcare organizations should integrate routine mental health screening with trauma-informed support systems in critical care units. Additionally, optimizing staffing models, enhancing leadership support, and ensuring access to confidential psychological services may improve nurse retention, patient safety, and the quality of care. Strengthening nurses’ well-being directly supports the priorities of Saudi Arabia’s Vision 2030, particularly those related to workforce development, quality of life, and healthcare system resilience. However, future longitudinal and mixed-method research is recommended to examine causal pathways and to evaluate culturally tailored interventions designed to sustain the well-being and performance of critical care nurses in Saudi Arabia.

## Figures and Tables

**Table 1:** Sociodemographic and professional characteristics of the study participants (N = 210).

Variable	*n* (%)
Age (years)	
25–34	127 (60.5)
35–44	53 (25.2)
≥45	30 (14.3)
Sex	
Male	34 (16.2)
Female	176 (83.8)
Education level	
Undergraduate and below	175 (83.3)
Postgraduate	35 (16.7)
Marital status	
Married	124 (59.1)
Single	86 (40.1)
Work unit	
ICU (medical, surgical, pediatric, neonatal)	128 (61)
Other critical units	82 (39)
Professional experience (years)	
1–2	36 (17.1)
3–5	57 (27.1)
6–10	39 (18.6)
>10	78 (37.1)
Shift length	
12 h shift	141 (67.1)
8 h shift	49 (23.3)
Other shift systems	20 (9.6)
Nurse-to-patient ratio	
1:1	36 (17.1)
1:2	103 (49.0)
1:3	38 (18.1)
≥1:4	33 (15.7)
Work region	
Eastern region (Al Sharqiyah)	121 (57.6)
Riyadh region	89 (42.4)
Monthly income (SAR)	
<6000	29 (13.8)
6000–8000	42 (20.0)
8000–12,000	95 (45.2)

**Table 2:** Descriptive statistics of study scales (N = 210).

Scale	Mean	Standard Deviation	Interpretation
PCL-5	27.44	17.5	Moderate level
CF	53.30	11.4	Moderate level
WHO-5	54.60	23.2	Moderate Level

CF, compassion fatigue; PCL-5, Post-Traumatic Stress Disorder Checklist; WHO-5, World Health Organization Well-Being Index.

**Table 3:** Mean score differences according to sociodemographic factors (N = 210).

Variables	PCL-5		CF		WHO-5	
	Mean ± SD	*p*-Value	Mean ± SD	*p*-Value	Mean ± SD	*p*-Value
Age						
25–34	28.1 ± 179	0.320	54.5 ± 11.4	0.161	13.0 ± 5.9	0.147
35–44	28.2 ± 16.9		51.5 ± 9.9		14.2 ± 5.2	
≥45	22.9 ± 16.8		51.3 ± 13.3		15.1 ± 5.9	
Education						
Undergraduate and below	27.2 ± 17.9	0.694	52.7 ± 11.1	0.123	13.6 ± 5.8	0.996
Postgraduate	28.5 ± 15.6		56.0 ± 12.3		13.6 ± 5.5	
Marital status						
Married	26.0 ± 16.3	0.154	51.8 ± 11.4	0.024	14.8 ± 5.5	<0.001
Single	29.5 ± 18.90		55.4 ± 11.1		11.9 ± 5.8	
Income						
<6000	20.9 ± 11.9	0.063	47.3 ± 9.3	<0.001	14.8 ± 5.7	0.012
6000–8000	25 ± 17.6		50.5 ± 10.4		15.8 ± 5.0	
8000–12,000	28.8 ± 19.1		54.0 ± 12.1		12.8 ± 6.1	
>12,000	31.0 ± 15.8		58.1 ± 9.6		12.5 ± 5.1	
Professional experience (years)						
1–2	32.5 ± 19.3	0.045	57.4 ± 9.9	0.059	9.6 ± 4.8	<0.001
3–5	24.4 ± 16.0		52.9 ± 11.5		14.8 ± 5.8	
6–10	31.5 ± 17.8		54.0 ± 10.3		12.5 ± 5.6	
>10	25.2 ± 16.9		51.2 ± 12.0		15.1 ± 5.3	
Nurse-to-patient ratio						
1:1	21.6 ± 13.8	0.162	49.3 ± 10.0	0.025	15.5 ± 5.2	0.105
1:2	28.5 ± 18.7		53.3 ± 11.4		13.3 ± 5.8	
1:3	27.7 ± 15.2		53.0 ± 11.4		13.9 ± 6.3	
≥1:4	30.1 ± 19.0		57.6 ± 11.5		12.2 ± 5.22	
Shift length						
12 h shift	26.6 ± 16.8	0.349	52.3 ± 10.7	0.081		
Other shift systems	29.0 ± 18.8		55.2 ± 12.5			

CF, compassion fatigue; PCL-5, Post-Traumatic Stress Disorder Checklist; SD, standard deviation; WHO-5, World Health Organization Well-Being Index.

**Table 4:** Correlation matrix for the main study variables (N = 210).

Variable	1	2	3
1-CF	1		
2-WHO-5	−0.396 ***	1	
3-PCL-5	0.719 ***	−0.387 ***	1

*** Correlation significant at the 0.001 level. CF, compassion fatigue; PCL-5, Post-Traumatic Stress Disorder Checklist; WHO-5, World Health Organization Well-Being Index.

**Table 5:** Multiple linear regression analysis predicting psychological well-being (WHO-5) among critical care nurses (N = 210).

Predictor	B^a^	SE	β^b^	t	*p*-Value	95% CI (Lower, Upper)
Age ^a^	0.244	0.959	0.021	0.254	0.800	−1.647, 2.134
Education level ^b^	−0.115	0.923	−0.009	−0.125	0.901	−1.935, 1.705
Professional experience ^c^	0.550	0.974	0.047	0.565	0.573	−1.371, 2.471
Monthly income ^d^	−1.579	0.874	−0.129	−1.806	0.072	−3.303, 0.145
Work region ^e^	−0.533	0.806	−0.045	−0.661	0.509	−2.122, 1.056
Shift length ^f^	0.191	0.826	0.015	0.231	0.818	−1.439, 1.820
Nurse-to-patient ratio ^g^	0.144	0.908	0.012	0.159	0.874	−1.645, 1.934
Work unit ^h^	−0.781	0.868	−0.066	−0.899	0.370	−2.494, 0.931
Compassion fatigue	−0.097	0.048	−0.20	−2.039	0.034 *	−0.191, −0.010
PTSD symptoms (PCL-5)	−0.073	0.030	−0.22	−2.413	0.017 *	−0.133, −0.015
Model summary: R^2^ = 0.21, F [8, 201] = 17.03, *p* < 0.01						

B^a^, unstandardized coefficient; β^b^, standardized coefficient (beta); CI, confidence interval; PCL-5, Post-Traumatic Stress; * *p* < 0.05. ^a^ Age = <35 years vs. ≥35 years; ^b^ Education level = Undergraduate and below vs. postgraduate; ^c^ Professional experience = ≤5 years vs. >5 years; ^d^ Monthly income = ≤8000 Riyal Saudi vs. >8000 Riyal Saudi; ^e^ Work region = eastern region vs. Riyadh region; ^f^ Shift length = 12 h shift vs. 8 h shift and others; ^g^ Nurse-to-patient ratio ≤ 1:2 vs. >1:2; ^h^ Work unit = Intensive Care Units vs. other critical units.

## Data Availability

The datasets generated can be obtained upon request by sending an email to alikariri@ksu.edu.sa due to privacy reasons.

## Abbreviations

The following abbreviations are used in this manuscript:

ANOVA	Analysis of variance
CI	Confidence interval
ICU	Intensive care unit
PCL-5	PTSD Checklist for DSM-5
ProQOL V5	Professional Quality of Life Scale Version 5
PTSD	Post-traumatic stress disorder
